# Butyrate Reducing Bone Mass Loss by Regulating the Expression of m6A Methyltransferase METTL3 in Implant‐Associated *Staphylococcus aureus* Osteomyelitis

**DOI:** 10.1111/jcmm.70683

**Published:** 2025-09-01

**Authors:** Chongkai Sun, Yuan Xu, Ziyue Peng, Xuyou Zhou, Zixuan Wang, Haoyang Wan, Bin Yu

**Affiliations:** ^1^ Department of Orthopaedics, Nanfang Hospital Southern Medical University Guangzhou China; ^2^ Guangdong Provincial Key Laboratory of Bone and Cartilage Regenerative Medicine, Nanfang Hospital Southern Medical University Guangzhou China

**Keywords:** butyrate, METTL3, N6‐methyladenosine, osteomyelitis, *Staphylococcus aureus*

## Abstract

*Staphylococcus aureus* ( *S. aureus* ) has been identified as a hindrance to osteoblast differentiation, thereby contributing significantly to the development of osteomyelitis. Consequently, exploring pharmaceutical interventions targeting osteoblast differentiation mediated by *S. aureus* may present a novel approach for treating osteomyelitis. It has been reported that N6‐methyladenosine (m6A) methylation is highly associated with infection. Moreover, studies continue to validate that short‐chain fatty acids play an important role in transcriptional modification and have been considered as a potential treatment for *S. aureus* infection. Our research aimed to examine the impact and underlying mechanism of butyrate, a short‐chain fatty acid, in reducing the inhibitory influence exerted by *S. aureus* on osteoblast differentiation. The concentration of butyrate beneficial to MC3T3‐E1 cell viability was screened by Cell Counting Kit‐8 (CCK8) assay. Reverse transcription‐quantitative PCR (RT‐qPCR), Western blotting, and alkaline phosphatase (ALP)staining were used to verify the osteogenic indexes and the expression levels of m6A methylation‐related proteins in MC3T3‐E1 cells infected with *S. aureus* at different time points. Besides, the same methods were used to verify the effects of butyrate stimulation and METTL3 knockdown on the osteogenic ability of MC3T3‐E1 cells. H&E staining, Goldner staining, and immunohistochemical staining were used to verify the effect of butyrate on mice with endo‐plant associated *S. aureus* osteomyelitis. The potential mechanisms of METTL3 and autophagy in MC3T3‐E1 cells were studied by Western blotting. In vitro experiments, we found that butyrate significantly enhanced the expression of osteogenic‐related genes down‐regulated by infection in MC3T3‐E1 cells induced by *S. aureus* , including RUNX2, OCN, and ALP. In addition, METTL3, an important m6A methyltransferase, was significantly up‐regulated in *S. aureus* ‐infected MC3T3‐E1 cells, but its expression could be down‐regulated by butyrate. Inhibiting the expression of METTL3 by siRNA could effectively rescue the osteogenic markers down‐regulated by *S. aureus* infection in MC3T3‐E1 cells, which was similar to the results of butyrate treatment. In vivo experiments had shown that butyrate could alleviate inflammation and osteogenic activity in implant‐associated osteomyelitis. The construction of bone marrow METTL3 low‐expression mice using siRNA also showed similar effects on osteogenic activity. Additionally, Western blotting confirmed that knocking down METTL3 rescued the autophagy imbalance caused by *S. aureus* infection in MC3T3‐E1 cells. In general, our research demonstrated that butyrate effectively alleviated the hindrance of osteoblast activity induced by *S. aureus* infection by suppressing the expression of METTL3, suggesting that butyrate may be a novel treatment for *S. aureus* osteomyelitis.

## 1 Introduction

Osteomyelitis is a commonly occurring infection that carries the risk of advancing to osteonecrosis and bone deterioration, causing local or systemic complications [1]. Once these symptoms persist for a duration exceeding 2 weeks, the condition can be classified as a chronic process [2]. The categorisation of osteomyelitis typically relies on the identification of the pathogens involved and the routes of infection. Osteomyelitis could be induced by various microorganisms, including bacteria, fungi and viruses. Gram‐positive bacteria are considered the main pathogenic strain of osteomyelitis, especially *S. aureus* , accounting for over 60% of osteomyelitis cases [3, 4]. Besides surgical resection of infected bone, the administration of local or systemic antibiotics has been identified as the main adjuvant therapy. Despite the ability of early antibiotic intervention to impede the advancement of infection being demonstrated, conventional antibiotic treatment strategies remain insufficient in addressing the osteolysis and bone deterioration resulting from the infection [5]. Consequently, the development of pharmaceutical agents capable of stimulating osteogenesis in cases of osteomyelitis holds considerable clinical importance.

RNA N6‐methyladenosine (m6A), a type of RNA modification existing in eukaryotes, is extensively involved with various biological and pathological processes including immunity, cancer and inflammation [6, 7]. The dynamic and reversible nature of m6A modification is facilitated by methyltransferase‐like 3 (METTL3), METTL14, and Wilms tumour 1‐related proteins [8, 9, 10], which form methyltransferase complexes, and m6A demethylases, including fat and obesity‐related proteins (FTO) and ALKB homologues 5 (ALKBH5) [11, 12]. Consequently, m6A exerts regulatory effects through interaction with readers [13, 14]. Several studies demonstrated a notable up‐regulation of METTL3 in the synovium of patients diagnosed with rheumatoid arthritis. Furthermore, suppressing the expression of METTL3 has been found to effectively impede the inflammatory response in fibroblast‐like synoviocytes [15]. Additionally, dysregulation of m6A‐related regulatory factors has been demonstrated in patients with osteosarcoma, which is associated with the metastasis and prognosis of disease [16]. However, there is a scarcity of research investigating the connection between m6A‐related regulatory factors and *S. aureus* infection.

Short‐chain fatty acids (SCFAs), such as acetate, propionate and butyrate, are predominantly produced through bacterial fermentation of dietary fibre within the gastrointestinal tract [17]. SCFAs play an important role in diverse physiological functions, including the regulation of gastrointestinal function, enhancing glucose tolerance, and anti‐inflammatory and metabolic effects on energy homeostasis [18, 19, 20]. The primary emphasis of this experiment lies in investigating the effects of butyrate. Previous studies have reported that butyrate indirectly influences the gene expression of Wnt10B, a crucial ligand in bone synthesis as well as T cells [21]. By regulating osteoclast‐specific signals and inhibiting histone deacetylase (HDAC) activity, butyrate exerts its inhibitory effect on osteoclast formation [22]. Despite the numerous reported advantages of butyrate in regulating bone metabolism, its specific effects on osteomyelitis remain unclear.

It is worth noting that butyrate can regulate biological activity by modulating m6A methylation. Research has shown that butyrate can mitigate the progression of polycystic ovary syndrome by downregulating the expression of METTL3 in KGN cells [23]. Furthermore, it has been demonstrated that METTL3 plays an important role in facilitating the development of colon cancer both in vitro and in vivo. Conversely, the expression of METTL3 and its associated cyclin E1 can be suppressed by butyrate, thereby preventing the progression of colorectal cancer [24].

We speculate that exploring the relationship between butyrate, METTL3, and osteomyelitis is of great significance. In this study, we aim to investigate the effects of butyrate therapy and changes in METTL3 expression on osteoblasts under infection conditions.

## 2 Materials and Methods

### 2.1 The Preparation of Butyrate

Butyrate is purchased from VETEC (Guangzhou, China, WXBD2346V). The butyrate was dissolved in the α‐MEM medium (Gibco, USA, C12571500BT) containing 10% fetal bovine serum (FBS, Gibco, USA, A5669701) and 1% penicillin–streptomycin solution (Gibco, USA, P1400) and diluted to the final concentration of 5 mg/mL. The whole dispensing process was completed in a sterile environment.

### 2.2 Bacterial Culture and Quantification

For the infection experiment, *S. aureus* was inoculated in 6 mL fresh trypsin soybean broth (TSB, Gibco, R112996) and incubated overnight at a rate of 200 rpm/min at 37°C. The bacteria were collected by centrifugation, washed twice with phosphate‐buffered saline (PBS, Gibco, USA, AAPR52‐500), and re‐suspended in 1 mL PBS. The concentration of *S. aureus* was adjusted to an OD of 0.5 at 600 nm, approximately equal to 1 × 10^8^ CFU/mL, then diluted to different concentrations for the osteomyelitis mice model or MC3T3‐E1 cell line.

### 2.3 Cell Culture and Differentiation

The preosteoblast line MC3T3‐E1 was utilised as a model for osteoblasts. The cells were cultured in a controlled environment with 95% air and 5% CO_2_ at a temperature of 37°C, in a medium supplemented with 10% FBS and 1% penicillin–streptomycin solution. To assess the impact of *S. aureus* and butyrate on osteoblast differentiation, MC3T3‐E1 cells were cultured in an osteogenic differentiation medium (containing 10% FBS, 10 mM HEPES, 50 μg/mL Lmurl ascorbic acid, and 5 mM β‐MEM) after infection with MOI = 10.

### 2.4 siRNA‐Transfection Treatment

The cells were transfected with siRNA targeting METTL3 (RIBOBIO, Guangzhou, China, SIGS0012383‐1). The siRNA sequences used were as follows: siMETTL3‐1 (CGTCAGTATCTTGGGCAAATT), siMETTL3‐2 (GCACCCGCAAGATTGAGTTAT) and siMETTL3‐3 (CCAGTCATAAACCAGATGAAA). Prior to transfection, the cells were seeded onto a cell pore plate. Transfection was conducted following the experimental instructions provided with the transfection kit. After 72 h, the medium was replaced with fresh medium and the cells were cultured for 7 days with or without MOI = 10 *S. aureus* infection. Subsequently, a series of experiments were performed on the cells.

### 2.5 CCK‐8 Assay

MC3T3‐E1 cells were cultured in 96‐well culture plates. The adherent cells were treated with the medium of 5 mg/mL butyrate solution or siRNA. The concentration of butyrate was 0, 10, 20, 50, 75, 100, 200 and 300 μg/mL. For each butyrate concentration or siRNA type, three parallel wells were set. After 24, 48 and 72 h of incubation, 10 μL CCK‐8 reagents (Beyotime, Shanghai, China, C0038) were added to each well and incubated in the incubator for 2 h. The absorbance at 450 nm (A450) was measured by I3X multifunctional enzyme labeling instrument (Germany).

### 2.6 ALP Staining

MC3T3‐E1 cells were cultured into 12‐well culture plates. After the cells began to adhere, the adherent cells were treated with butyrate solution or siRNA. The concentration of butyrate was 10, 20 μg/mL and cultured in osteogenic differentiation medium to induce differentiation. After 7 days of treatment, the cells were fixed with paraformaldehyde for 30 min and washed with PBS 3 times. According to the manufacturer's instructions, the cells were stained with the ALP staining kit (Beyotime, Shanghai, China, P0321S). Staining was observed and recorded by a scanner and BX63 inverted microscope (Olympus, Tokyo, Japan).

### 2.7 Western Blotting

The cells were lysed with RIPA buffer containing protease inhibitors. The concentration was detected by BCA kit. The same amount of protein (30 μg) was added to 10% or 12.5% polyacrylamide gel by SDS‐PAGE, and then transferred to the PVDF membrane. 30 min was blocked with a fast‐blocking liquid (Epizyme, Shanghai, China, PS108P) solution at room temperature, and then incubated overnight with the first antibody of the target protein. On the second day, the membrane was washed with TBST 3 times, each 10 min, then further incubated with anti‐rabbit secondary antibody at room temperature for 1 h. Rinse with TBST 3 times, each time 10 min. All imprints were developed using ECL chromogenic solution (BioSharp, Canada, BL520A). Use BLT chemiluminescence device (Shanghai, China) images.

### 2.8 qRT‐PCR

RNA extraction using RNA extraction Kit (TIANGEN, Beijing, China, Y2102) was used to obtain cellular RNA; lysate reagent was used to isolate total RNA. After that, RNA was precipitated with isopropanol. After the supernatant was completely removed, RNA washing buffer (anhydrous ethanol) was added and centrifuged. After centrifugation, eluent and rinse solution were added to eluate and rinse RNA. The concentration and quality of RNA samples were determined by spectrophotometer. Then reverse transcription of RNA into cDNA was performed by PrimeScriptTM RT kit (TaKaRa, Dalian, China, RR037Q). Use iQ5 (Bio‐Rad, Hercules, CA, USA) and SYBR Green Pro Taq HS (AG, Hunan, China, AG11701) to enter qPCR. GAPDH was used as an internal reference gene, and the relative expression of the gene was standardised as GAPDH and processed by the 2^−∆∆t^ method. The sequence of primers used in the experiment is shown in Table 1.

**TABLE 1:** The primer sequence was used in qRT‐PCR.

Gene	Primer sequence
METTL3	Forward: 5′‐CTTTAGCATCTGGTCTGGGCT‐3′ Reverse: 5′‐TCCTTGTCGTCTCGTTCTTCC‐3′
METTL14	Forward: 5′‐TCTGGAAAACTGCCTTTGGAT‐3′ Reverse: 5′‐AAATGCTGGACCTGGGATGAT‐3′
FTO	Forward: 5′‐ACTGGTTTTCCGAGAGGCTG‐3′ Reverse: 5′‐GTGAGCACGTCTTTGCCTTG‐3′
ALKBH5	Forward: 5′‐ACCACCAAACGGAAGTACCAG‐3′ Reverse: 5′‐TGCAGAGAAGTCGGTCCTACT‐3′
RUNX2	Forward: 5′‐TGGTTACTGTCATGGCGGGTA‐3′ Reverse: 5′‐TCTCAGATCGTTGAACCTTGCTA‐3′
ALP	Forward: 5′‐ACCACCACGAGAGTGAACCA‐3′ Reverse: 5′‐CGTTGTCTGAGTACCAGTCCC‐3′
OCN	Forward: 5′‐CACTCCTCGCCCTATTGGC‐3′ Reverse: 5′‐CCCTCCTGCTTGGACACAAAG‐3′
GAPDH	Forward: 5′‐GCAAAGTGGAGATTGTTGCC‐3′ Reverse: 5′‐TGGAAGATGGTGATGGGCTT‐3′

### 2.9 Animal

Eight‐week‐old male C57BL/6 mice were purchased from the Experimental Animal Center of Southern Medical University (Guangzhou, China). The mice were raised under specific pathogen‐free conditions of 24°C–26°C, the light/dark cycle was 12 h, and food and water could be obtained at will. All interventions and animal care procedures were carried out by the animal surgery guidelines and policies provided by Southern Hospital. The mice were randomly divided into Ctrl group, *S. aureus* group, butyrate group and siMETTL3 group. The butyrate feeding group was fed with water containing butyrate at the concentration of 50 mM 2 weeks before infection [25]. The mouse model of osteomyelitis was established by inoculating the femur with *S. aureus* . Soak a 2 mm long sterile stainless steel needle (diameter 0.3 mm) in PBS with a concentration of 1 × 10^6^ CFU/mL *S. aureus* for 1 h in advance. After the mice were anaesthetised by intraperitoneal injection of tribromoethanol, the incision was made along the dorsal side of the femur to expose the bone surface. Drill a hole in the cortical bone of the middle shaft of the femur with an electric drill without penetrating the other side. For the control group, the sterile stainless steel needle was inserted into the bone marrow cavity through the hole with saline injection as a placebo. In the siMETTL3 group, siMETTL3 containing 0.1 nmol was injected into the bone marrow cavity by syringe (diameter 0.35 mm). Next, insert the bacteria‐soaked stainless steel needle into the bone marrow cavity. The incisions were closed with 5–0 sutures. Two weeks after the operation, the animals with cervical dislocation were sacrificed and femurs were collected for further experiment.

### 2.10 Histochemistry and Immunohistochemistry

The femur was fixed overnight with 4% paraformaldehyde, decalcified in 10% EDTA (pH = 8, Solarbio, Beijing, China, E8040) for 1 week, and finally embedded in paraffin. Sagittal sections with a thickness of 4 μm were cut longitudinally and stained. Two of every ten consecutive slices were stained with haematoxylin and eosin (Solarbio, Beijing, China, G1120). Immunohistochemical staining was performed according to the standard scheme. After dewaxing and rehydration, the slices were incubated with the first antibody of the target gene at 4°C overnight. After washing in PBS, the slices were incubated with secondary antibodies, and then incubated with avidin‐biotinylated horseradish peroxidase complex. Peroxidase activity was revealed by DAB (ZSGB‐BIO, Beijing, China, ZL1‐9017).

### 2.11 Goldner Trichromatic Staining

Goldner trichromatic staining (Solarbio, Beijing, China, g3550) was used to evaluate the activity of osteoblasts. The tissue sections were dewaxed in xylene, rehydrated with gradient ethanol, and then stained according to the manufacturer's instructions using the Goldner trichromatic staining kit. The staining results showed that the new bone was orange‐red, the osteoid was purple, the mature bone was green, and the nucleus was blue‐grey. The area of new bone dyed orange‐red was calculated using Image Pro Plus 6.0.1.

### 2.12 Statistical Analysis

The data were analysed by student t‐test or one‐way analysis of variance (as the case may be) using GraphPad version 7.03 (GraphPad Software Inc., LaJolla, CA). The results were expressed as the average ± SEM; *p* < 0.05 was set to be statistically significant.

## 3 Result

### 3.1 Low Concentration Butyrate Is Beneficial for the Proliferation of MC3TC‐E1 Cells

**FIGURE 1:**
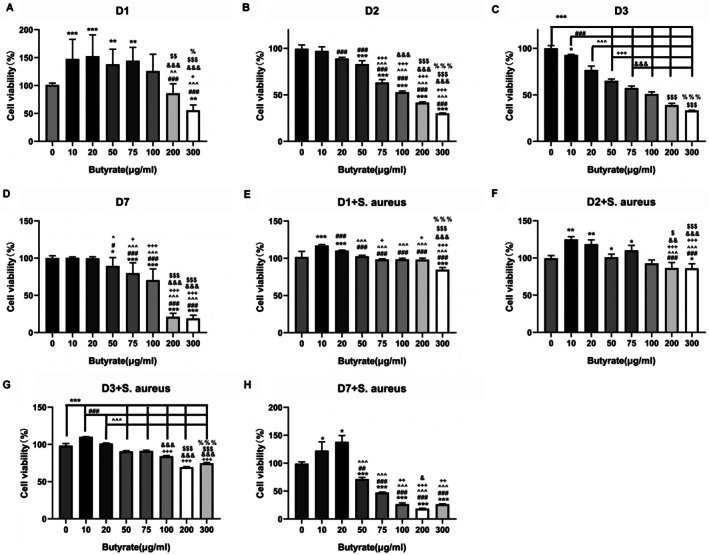
The effect of butyrate on the viability of MC3T3‐E1 cells and the inhibition of inflammation induced by *S. aureus* . CCK8 assay was used to determine the effect of butyrate on the viability of MC3T3‐E1 cells and *S. aureus* treated MC3T3‐E1 cells at 24, 48, 72 h and 7 days. *n* = 3 in each group. *p < 0.05, **p < 0.01, ***p < 0.001 versus 0 μg/mL. ^#^ *p* < 0.05, ^##^ *p* < 0.01, ^###^ *p* < 0.001 versus 10 μg/mL. ^*p* < 0.05, ^^*p* < 0.01, ^^^*p* < 0.001 versus 20 μg/mL. ^+^ *p* < 0.05, ^++^ *p* < 0.01, ^+++^ *p* < 0.001 versus 50 μg/mL. ^&^ *p* < 0.05, ^&&^ *p* < 0.01, ^&&&^ *p* < 0.001 versus 75 μg/mL. ^$^ *p* < 0.05, ^$$^ *p* < 0.01, ^$$$^ *p* < 0.001 versus 100 μg/mL. ^%^ *p* < 0.05, ^%%^ *p* < 0.01, ^%%%^ *p* < 0.001 versus 200 μg/mL. *S. aureus* , *Staphylococcus aureus* .

CCK8 assay was initially used to assess the effect of different concentrations of butyrate on the proliferation of MC3T3‐E1 cells, either with or without *S. aureus* infection. After 24 h treatment of 10–75 μg/mL butyrate, the proliferation of MC3T3‐E1 cells showed slightly increased compared to the control group. However, higher concentrations (≥ 200 μg/mL) of butyrate treatment showed an adverse effect (Figure 1A). As the time of treatment extended to 48, 72 and 168 h, except for those groups treated with 10 and 20 μg/mL, other groups consistently revealed inhibition on cell proliferation compared to the control group (Figures 1A–D), which suggested potential cytotoxicity of butyrate at high concentration. With the infection of *S. aureus* , treatment of 10 and 20 μg/mL of butyrate remained protective of cell viability from 24 h to 7 days (Figures 1E–H). However, butyrate exceeding 20 μg/mL was disabled to maintain cell proliferation against *S. aureus* , and inhibition of cell proliferation was found to aggravate as the concentration of butyrate increased (Figures 1H). Therefore, a concentration of butyrate lower than 20 μg/mL was demonstrated to be beneficial for the viability of MC3T3‐E1 cells, and 10 and 20 μg/mL of butyrate were selected for further in vitro experiments.

### 3.2 Butyrate Saves the Decrease of Osteogenic Capability of MC3T3‐E1 Cells Induced by *S. aureus* Infection

**FIGURE 2:**
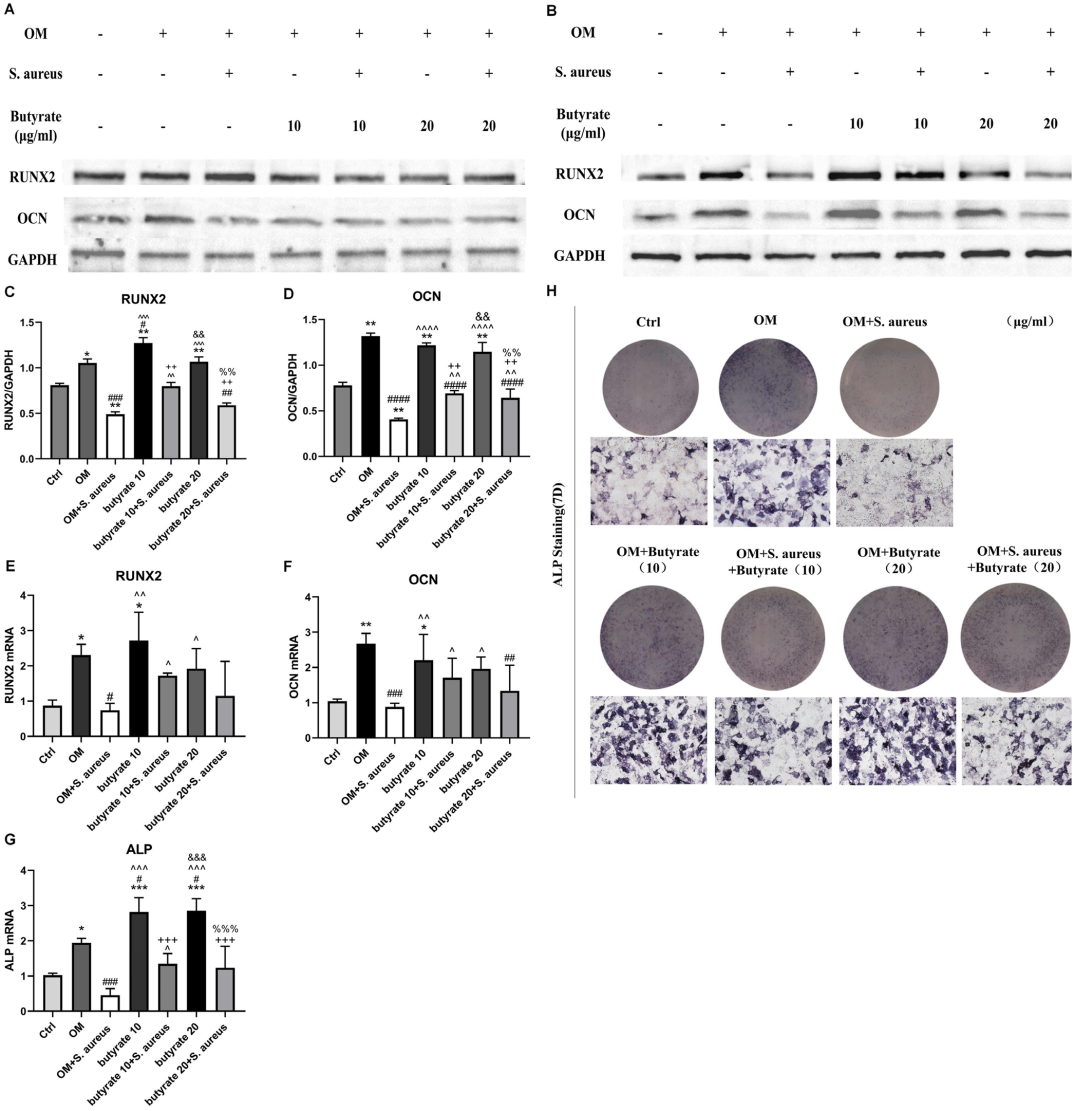
A certain concentration of butyrate could increase the down‐regulated osteogenic capability (A) The protein levels of osteogenic indexes (RUNX2, OCN) were analysed by Western blotting after 0, 10 and 20 μg/mL butyrate treatment with or without *S. aureus* infection for 24 h. (B) The protein levels of osteogenic indexes (RUNX2, OCN) were analysed by Western blotting after 0,10 and 20 μg/mL butyrate treatment with or without *S. aureus* infection for 7 days. (C, D) Protein quantification of (B) by ImageJ. *n* = 3 in each group. (E–G) qPCR analysis of osteogenic indexes (RUNX2, OCN and ALP) 7 days after 0,10 and 20 μg/mL butyrate treatment with or without *S. aureus* infection. (H) Scanning and microscopic images of MC3T3‐E1 cells treated with and without *S. aureus* infection at 10 and 20 μg/mL butyrate for 7 days after ALP staining. *p < 0.05, **p < 0.01, ***p < 0.001 versus Ctrl. ^#^ *p* < 0.05, ^##^ *p* < 0.01, ^###^ *p* < 0.001 versus OM. ^*p* < 0.05, ^^*p* < 0.01, ^^^*p* < 0.001 versus OM + *S. aureus* .^+^ *p* < 0.05,^++^ *p* < 0.01,^+++^ *p* < 0.001 versus Butyate 10. ^&^ *p* < 0.05, ^&&^ *p* < 0.01, ^&&&^ *p* < 0.001 versus Butyrate 10 + *S. aureus* . ^%^ *p* < 0.05,^%%^ *p* < 0.01,^%%%^ *p* < 0.001 versus Butyrate 20. *S. aureus* , *Staphylococcus* *aureus; RUNX2, Runt*‐related transcription factor 2; OCN, osteocalcin; ALP, Alkaline Phosphatase; OM, Osteogenic medium; qPCR, quantitative PCR.

Previous research has indicated that osteomyelitis caused by *S. aureus* can result in a decline in osteogenic activity. We hypothesise that butyrate can prevent the decline in osteogenic index. Following infection of MC3T3 cells with *S. aureus* at a MOI = 10, the osteogenesis‐related genes were assessed at 24 h and 7 days. The results revealed no significant change in the osteogenic capability within 24 h (Figure 2A), but a significant decrease in the osteogenic genes RUNX2, OCN and ALP was observed after 7 days of infection. Following the introduction of either 10 or 20 μg/mL butyrate subsequent to *S. aureus* infection, a notable up‐regulation of the osteogenic capability was observed (Figures 2B–G). Additionally, the ALP mineralised nodule staining provided further evidence of the osteogenic impact of butyrate in the presence of infection (Figure 2H). Interestingly, the osteogenic capacity of MC3T3‐E1 cells was not enhanced by butyrate in the absence of *S. aureus* infection. In summary, the findings suggest that butyrate could potentially augment the osteogenic ability of MC3T3‐E1 cells that have been down‐regulated due to infection.

### 3.3 Butyrate Reversed Up‐Regulation of METTL3 Expression in MC3T3‐E1 Cells Induced by *S. aureus* Infection

**FIGURE 3:**
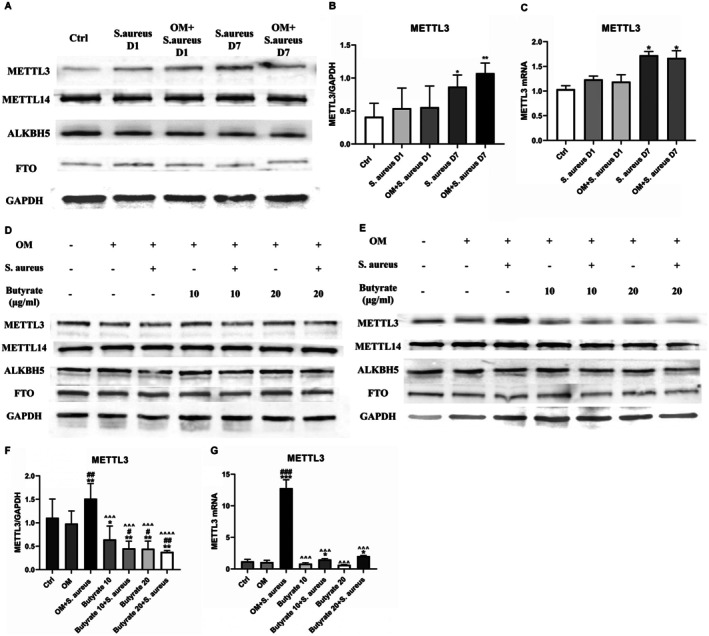
After 7 days of *S. aureus* infection, METTL3 levels were up‐regulated in MC3T3‐E1 cells, which could be reversed by low concentration butyrate treatment. (A) The protein levels of m6A methylation‐related genes (METTL3, METTL14, FTO and ALKBH5) in MC3T3‐E1 cells treated with or without *S. aureus* infection in osteogenic medium for 24 h or 7 days were analysed by Western blotting. (B) Protein quantification of (A) by ImageJ. *n* = 3 in each group. *p < 0.05, **p < 0.01 versus Ctrl. (C) qPCR analysis of m6A methyltransferase METTL3 in MC3T3‐E1 cells treated with or without *S. aureus* infection and with or without osteogenic medium for 24 h or 7 days. *p < 0.05 versus Ctrl. (D) The protein levels of m6A methylation‐related genes (METTL3, METTL14, FTO, ALKBH5) in MC3T3‐E1 cells treated with 0, 10 or 20 μg/mL butyrate groups with or without *S. aureus* infection for 24 h were analysed by Western blotting. (E) The protein levels of m6A methylation‐related genes (METTL3, METTL14, FTO, ALKBH5) in MC3T3‐E1 cells treated with 0, 10 or 20 μg/mL butyrate groups with or without *S. aureus* infection for 7 days were analysed by Western blotting. (F) Protein quantification of (E) by ImageJ. *n* = 3 in each group. (G) qPCR analysis of m6A methyltransferase METTL3 in MC3T3‐E1 cells treated with 0, 10 or 20 μg/mL butyrate with or without *S. aureus* infection for 7 days. *p < 0.05, **p < 0.01, ***p < 0.001 versus Ctrl. ^#^ *p* < 0.05, ^##^ *p* < 0.01, ^###^ *p* < 0.001 versus OM. ^*p* < 0.05, ^^*p* < 0.01, ^^^*p* < 0.001 versus OM + *S. aureus* . ^+^ *p* < 0.05, ^++^ *p* < 0.01, ^+++^ *p* < 0.001 versus Butyate 10. ^&^ *p* < 0.05, ^&&^ *p* < 0.01, ^&&&^ *p* < 0.001 versus Butyrate 10 + *S. aureus* . ^%^ *p* < 0.05, ^%%^ *p* < 0.01, ^%%%^ *p* < 0.001 versus Butyrate 20. *S. aureus* , *Staphylococcus aureus* ; OM, Osteogenic media; m6A, N6 methyladenosine; METTL3, methyltransferase‐like 3; METTL14, methyltransferase‐like 14; ALKBH5, AlkB homologue 5; FTO, Fat mass and obesity associated gene; qPCR, quantitative PCR.

The regulation of various biological activities by m6A methylation prompts speculation regarding its potential significance in osteomyelitis. To investigate the potential involvement of m6A methylation in the regulation of osteoblast formation during infection, the expression of m6A methylation‐related genes was assessed during the process of osteogenic differentiation, both with and without treatment with *S. aureus* . The findings of our study show that the level of m6A methylation‐related genes in MC3T3‐E1 cells infected with *S. aureus* remained unchanged after 24 h, regardless of osteoblast differentiation. However, after 7 days of infection, both the protein and mRNA levels of METTL3 exhibited an increase, while the expression of other genes did not show significant alterations (Figures 3A–C).

As mentioned earlier, butyrate can affect the occurrence and development of diseases by regulating the expression of METTL3. Consequently, we hypothesised that the administration of butyrate can mitigate the decline in the osteogenic capability of MC3T3‐E1 cells following *S. aureus* infection, potentially through the regulation of METTL3 expression. In the next experiment, MC3T3‐E1 cells were infected with *S. aureus* and subsequently treated with 10/20 μg/mL butyrate. Following a 24‐h treatment, no significant alteration in the expression level of m6A methylation‐related protein was observed (Figure 3D). However, after a 7‐day treatment, the addition of butyrate effectively decreased the expression of METTL3 in the infected group (Figures 3E–G). Notably, the expression pattern of METTL3 exhibited an inverse relationship with that of osteogenic markers. These findings suggest that butyrate can down‐regulate the expression of METTL3 in MC3T3‐E1 cells induced by *S. aureus* infection, thus improving osteogenic activity.

### 3.4 Down‐Regulation of METTL3 Through RNA Interference Up‐Regulates Osteogenic Activity of MC3T3‐E1 Cells Against *S. aureus* Infection

**FIGURE 4:**
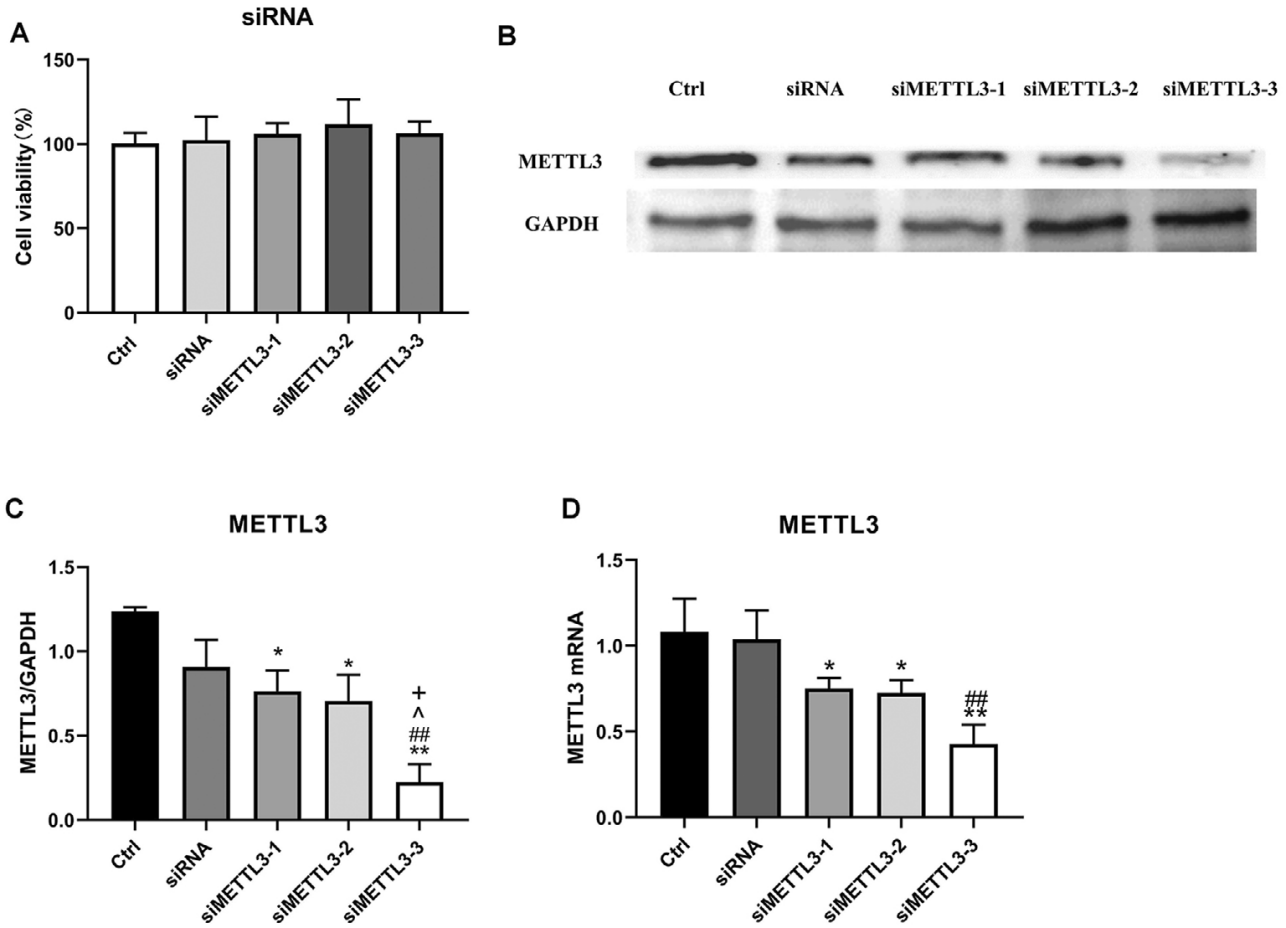
Screening of siMETTL3 (A) CCK8 assay was used to determine the effect of siMETTL3 on the viability of MC3T3‐E1 cells. (B) The protein level of m6A methyltransferase METTL3 in MC3T3‐E1 cells treated with three different siMETTL3 and siRNA were analysed by Western blotting. (C) Protein quantification of (B) by ImageJ. *n* = 3 in each group. (D) qPCR analysis of m6A methyltransferase METTL3 of MC3T3‐E1 cells treated with three different siMETTL3 or siRNA. *p < 0.05, **p < 0.01 versus Ctrl. ^##^ *p* < 0.01 versus siRNA. ^*p* < 0.05 versus siMETTL3‐^+^1. *p* < 0.05 versus siMETTL3‐2. siRNA, small interfering RNA; si‐METTL3, siRNA targeting METTL3; METTL3, methyltransferase‐like 3; qPCR, quantitative PCR.

**FIGURE 5:**
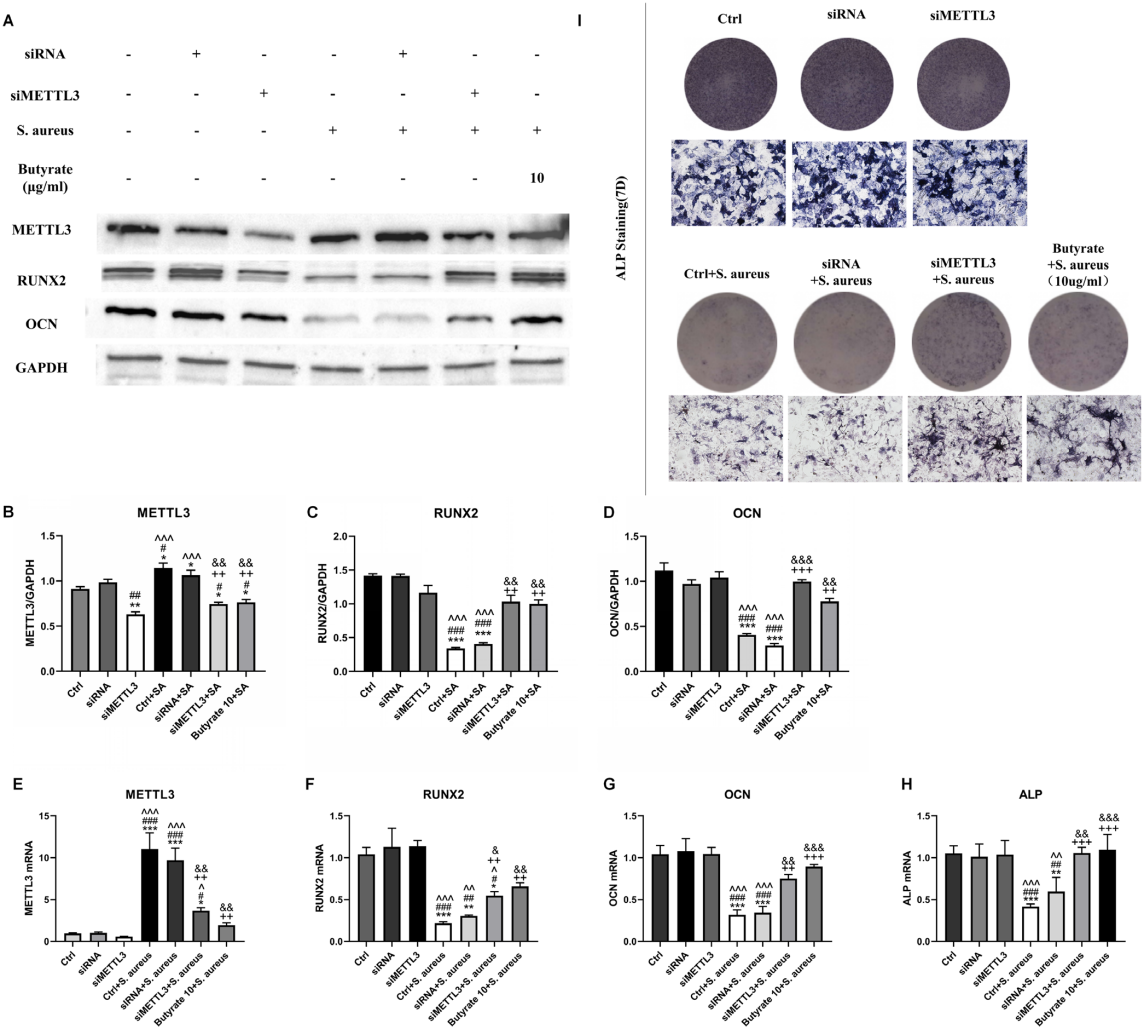
Silencing METTL3 restored the osteogenic index down‐regulated by *S. aureus* infection in MC3T3‐E1 cells. (A) The protein levels of m6A methyltransferase METTL3, osteogenic index RUNX2 and OCN in MC3T3‐E1 cells treated with or without *S. aureus* , siMETTL3, siRNA and butyrate were analysed by Western blotting. (B–D) Protein quantification of (A) by ImageJ. *n* = 3 in each group. (E–H) qPCR analysis of m6A methyltransferase METTL3, osteogenic index RUNX2, OCN and ALP of MC3T3‐E1 cells treated with or without *S. aureus* , siMETTL3, siRNA and butyrate for 7 days. *n* = 3 in each group. (I) Scanning and microscopic images of MC3T3‐E1 cells treated with or without *S. aureus* , siMETTL3, siRNA and butyrate for 7 days after ALP staining. *p < 0.05, **p < 0.01, ***p < 0.001 versus Ctrl. ^#^ *p* < 0.05, ^##^ *p* < 0.01, ^###^ *p* < 0.001 versus siRNA. ^*p* < 0.05, ^^*p* < 0.01, ^^^*p* < 0.001 versus siMETTL3. ^+^ *p* < 0.05, ^++^ *p* < 0.01, ^+++^ *p* < 0.001 versus Ctrl + *S. aureus*. ^&^ *p* < 0.05, ^&&^ *p* < 0.01, ^&&&^ *p* < 0.001 versus siRNA+ *S. aureus* . ^%^ *p* < 0.05, ^%%^ *p* < 0.01, ^%%%^ *p* < 0.001 versus siMETTL3 + *S. aureus* . siRNA, small interfering RNA; si‐METTL3, siRNA targeting METTL3; METTL3, methyltransferase‐like 3; *S. aureus* , *Staphylococcus aureus; m6A*, N6‐methyladenosine; RUNX2, Runt‐related transcription factor 2; OCN, osteocalcin; ALP, alkaline phosphatase; qPCR, quantitative PCR.

In order to investigate the relationship between METTL3 in MC3T3‐E1 cells and the osteogenic response to *S. aureus* infection, siRNA targeting METTL3 was utilised to decrease the expression of METTL3 in MC3T3‐E1 cells. Cell viability of MC3T3‐E1 cells was assessed using CCK8 detection, and it was determined that the presence of three specific METTL3 siRNAs did not affect cell proliferation at 24, 48 and 72 h (Figure 4A). Simultaneously, the outcomes obtained from Western blotting and qPCR analyses demonstrated a significant reduction in METTL3 expression in MC3T3‐E1 cells upon specific METTL3 siRNA treatment. Notably, siMETTL3‐3 exhibited the most effective silencing effect (Figures 4B,C), thus it was selected as the targeted siRNA for subsequent experiments. Subsequently, to investigate the alterations in osteogenic markers in *S. aureus* ‐infected cells following METTL3‐siRNA transfection, Western blotting and qPCR analyses were conducted. These findings demonstrate that the expression of osteogenic genes was up‐regulated in METTL3‐siRNA‐transfected MC3T3‐E1 cells when treated with *S. aureus* , similar to the response observed with butyrate treatment. However, there was no significant difference in the osteogenic expression of METTL3‐siRNA transfected osteoblasts in the absence of *S. aureus* infection (Figures 5A–I). These results indicate that inhibition of METTL3 can enhance the osteogenic activity of MC3T3‐E1 cells in the presence of *S. aureus* infection.

### 3.5 Butyrate Reduces Bone Loss and Inflammation of Bone Tissue Caused by *S. aureus* Infection

**FIGURE 6:**
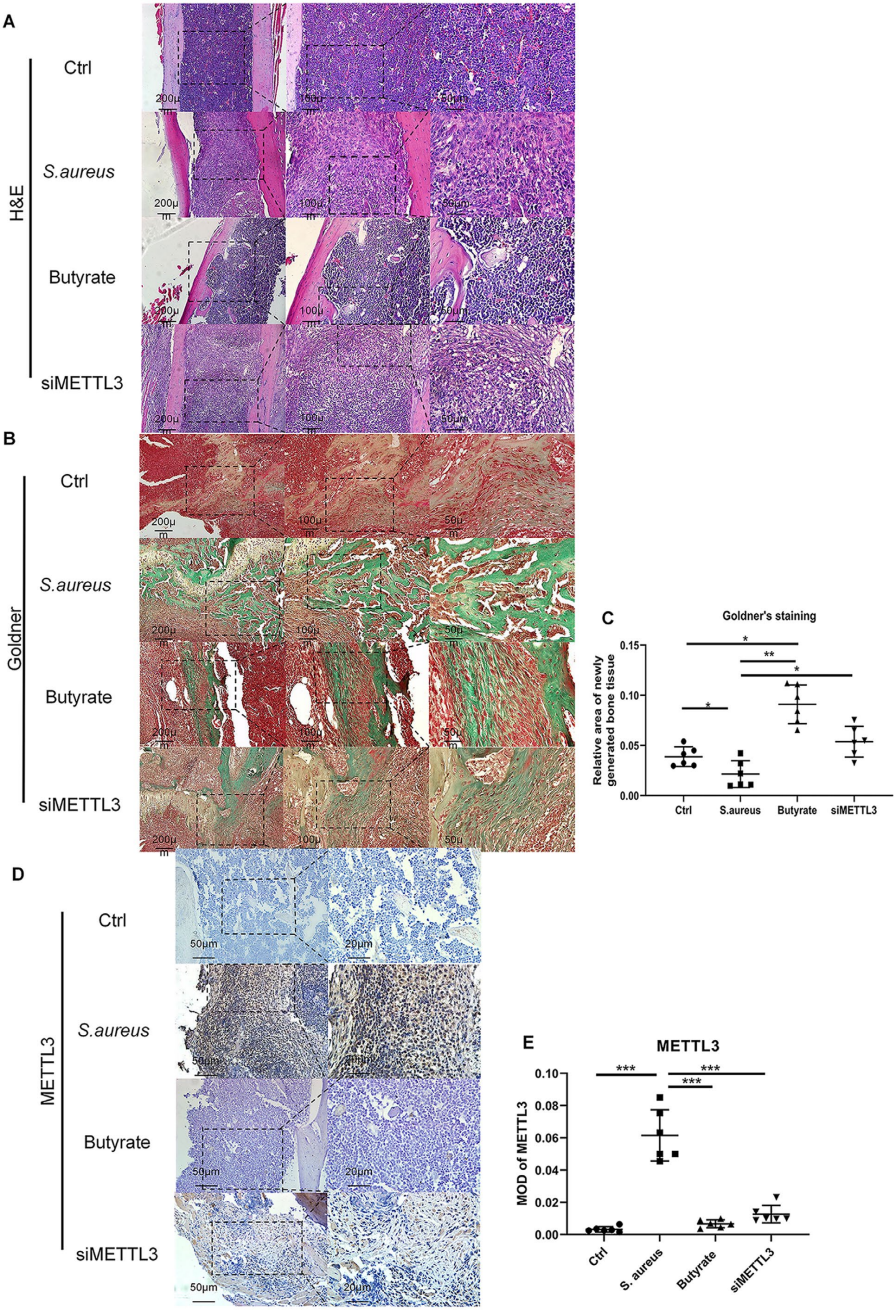
Mouse femur H&E staining, Goldner trichrome staining results, and METTL3 immunohistochemistry results revealed the anti‐inflammatory, bone promoting, and METTL3 inhibiting effects of butyrate in a plant associated *Staphylococcus aureus* osteomyelitis mouse model (A) H&E staining was performed on the bones of Ctrl group, *S. aureus* group, butyrate group and siMETTL3 group. The scale represents 200, 100 and 50 μm from left to right, respectively. (B) Goldner staining was performed on the bones of Ctrl group, *S. aureus* group, butyrate group and siMETTL3 group. The scale represents 200, 100 and 50 μm from left to right, respectively. (C) The relative area ratio of new bone to mature bone was analysed by Image Pro Plus 6.0 for (B). *n* = 6 in each group. *p < 0.05, **p < 0.01. (D) Representative images of METTL3 immunohistochemical staining of bone in Ctrl group, *S. aureus* group, butyrate group and siMETTL3 group. The scale from left to right represents 50 and 20 μm, respectively. (E) Quantitative analysis of METTL3 was performed by Image Pro Plus 6.0. The immunohistochemical staining average optical density value (MOD) of immunohistochemical staining was used for quantitative analysis of (D). *n* = 6 in each group. *p < 0.05, **p < 0.01, ***p < 0.001. si‐METTL3, siRNA targeting METTL3; *S. aureus* , *Staphylococcus aureus* ; METTL3, methyltransferase‐like 3.

Subsequently, a mouse model of osteomyelitis was successfully established, and the histological results further confirmed the differences in the severity of osteomyelitis among the groups. In comparison to the Ctrl group, the *S. aureus* group exhibited characteristic manifestations of osteomyelitis, such as intraosseous acute and chronic inflammation, osteonecrosis, and the formation of abscesses due to the accumulation of inflammatory cells. Conversely, the butyrate group did not display any discernible signs of inflammation. Intriguingly, the siMETTL3 group did not exhibit a reduction in the extent of inflammation (Figure 6A). These findings indicate that butyrate can inhibit inflammation in the body to support bone infection control. Although new bone formation was observed in all experimental groups, there were notable variations between the groups. Goldner staining allowed for the identification of both mature (green) and immature (red) bone tissue, providing a means to assess osteogenic activity. In the *S. aureus* group, only a small amount of fibrous tissue was formed to fill the defect, whereas the butyrate group and siMETTL3 group exhibited significantly enhanced bone formation, with most bone defects filled with newly generated bone tissue. The new bone tissue in both the butyrate group and siMETTL3 group exhibited extensive coverage of the defect, predominantly consisting of mature bone tissue that appeared green. Furthermore, quantitative analysis of the new bone area indicated that both the butyrate group and siMETTL3 group demonstrated enhanced osteogenic capability compared to the *S. aureus* group. Notably, no significant disparity was observed in the measurement of bone regeneration between the butyrate group and the siMETTL3 group (Figures 6B,C).

Subsequently, immunohistochemistry will be employed to ascertain the expression of METTL3 in the femurs of four distinct cohorts of mice, thereby corroborating our in vitro experimental outcomes. The expression of METTL3 (as indicated by brown staining) in the *S. aureus* group mice was significantly higher than that in the ctrl group mice and can be determined through quantitative analysis. Moreover, the expression level of METTL3 was significantly diminished in both the butyrate group and the siMETTL3 group, as evidenced by a substantial reduction in the area exhibiting brown staining (Figures 6D,E). This observation suggests the inhibitory impact of butyrate on METTL3 expression in osteomyelitis mice.

**FIGURE 7:**
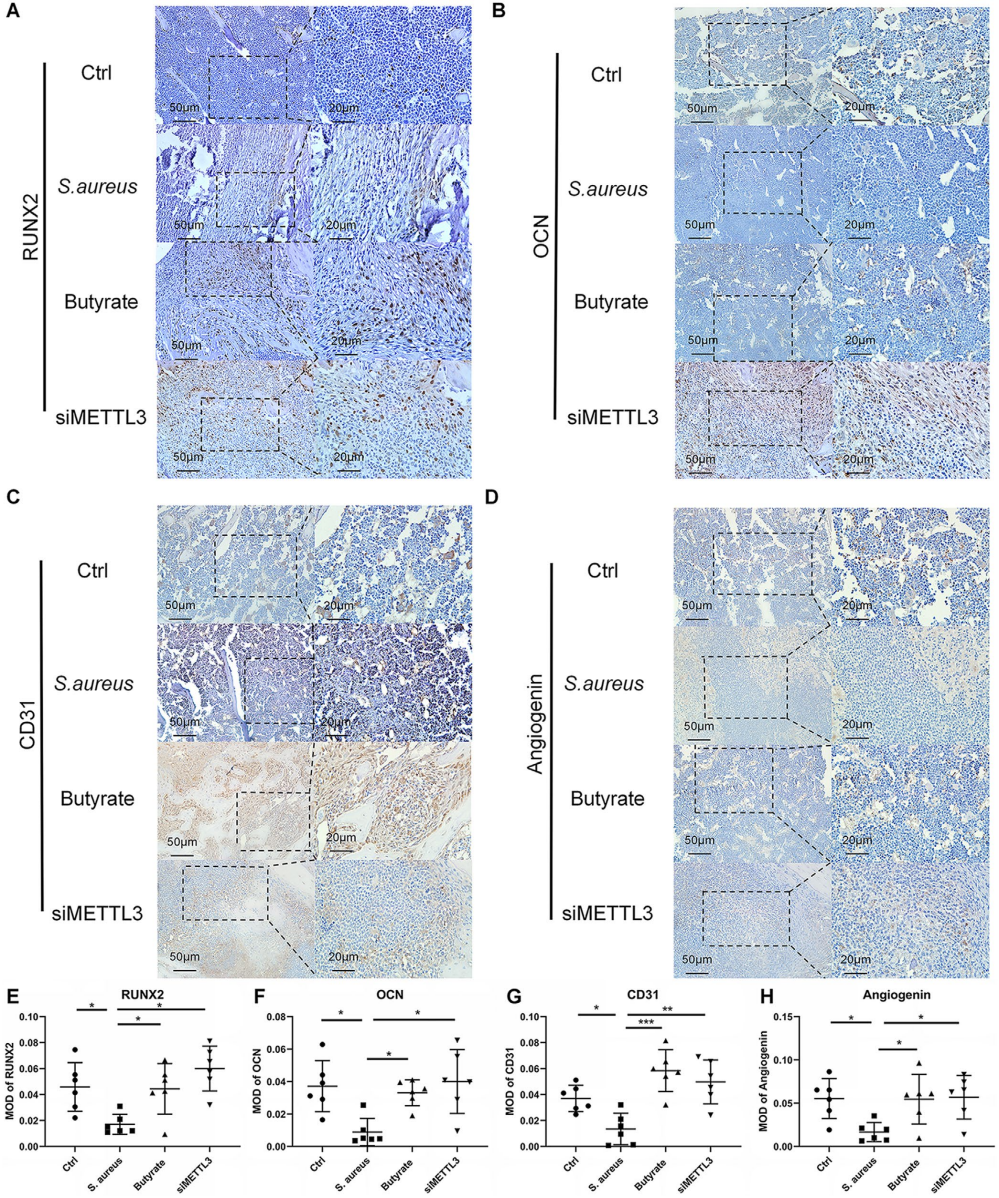
Immunohistochemical results of RUNX2, OCN, CD31 and Angiogenin in the femur of mice (A–D) Representative images of RUNX2, OCN, CD31 and Angiogenin immunohistochemical staining of bone in the Ctrl group, *S. aureus* group, Butyrate group and siMETTL3 group. The scale from left to right represents 50 and 20 μm, respectively. (E–H) Quantitative analysis of RUNX2, OCN, CD31 and Angiogenin was performed by Image Pro Plus 6.0. The immunohistochemical staining average optical density value (MOD) of immunohistochemical staining was used for quantitative analysis of (A–D). *n* = 6 in each group. *p < 0.05, **p < 0.01, ***p < 0.001. si‐METTL3, siRNA targeting METTL3; *S. aureus* , *Staphylococcus aureus* ; RUNX2, Runt‐related transcription factor 2; OCN, osteocalcin.

The expression of OCN and RUNX2, two indicators of osteoblast differentiation, was examined to confirm the changes of bone formation in the defect area. The findings revealed that the expression of Runx2 and OCN (as indicated by brown staining) was higher in both the butyrate group and siMETTL3 group compared to the *S. aureus* group. Moreover, the quantitative analysis demonstrated a significant increase in the expression of RUNX2 and OCN in the butyrate group and siMETTL3 group compared to the *S. aureus* group. No statistically significant disparity was observed in the expression level of Ocn or Runx2 within the defect area between the butyrate group and the siMETTL3 group (Figures 7A,B,E,F).

The vascularization process plays a crucial role in the development of bone within newly formed tissues [26, 27]. To assess the degree of vascularization in the regenerated bone tissue, the vascular markers CD31 and Angiogenin were examined. The expression levels of vascular markers in the butyrate group and siMETTL3 group were found to be higher than those in the *S. aureus* group, suggesting the development of more mature vascular tissue. Furthermore, quantitative analysis revealed a significant increase in both the density and number of vessels in the butyrate group and siMETTL3 group compared to the *S. aureus* group. However, no significant difference was observed between the butyrate group and siMETTL3 group in terms of vascular density and number (Figures 7C,D,G,H).

### 3.6 METTL3 May Be Involved in Regulating Autophagy in MC3T3‐E1 Cells

**FIGURE 8:**
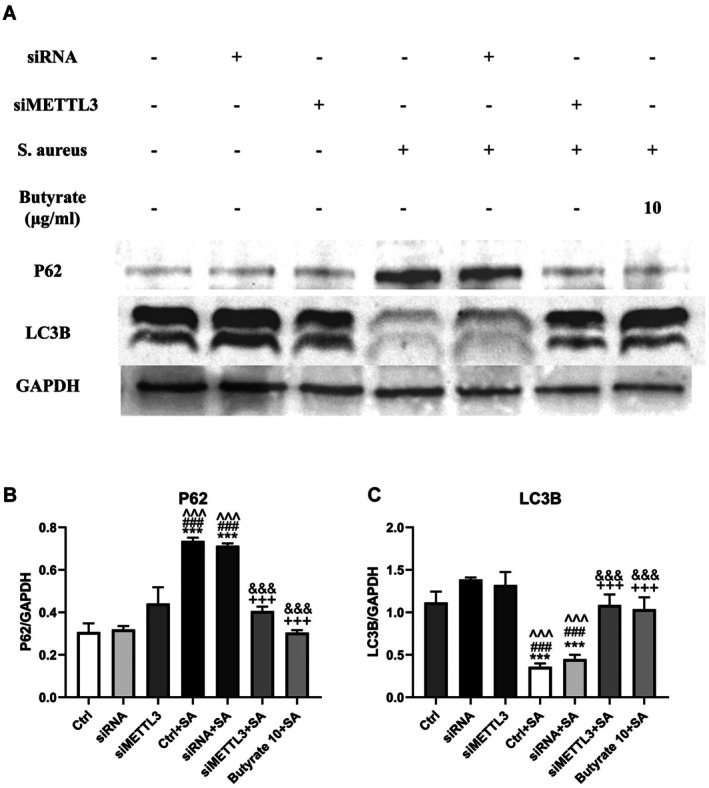
Silent METTL3 can save autophagy of MC3T3‐E1 cells damaged by *S. aureus* infection (A) The protein levels of P62 and LC3B in MC3T3‐E1 cells treated with or without *S. aureus* , siMETTL3, siRNA and butyrate for 7 days were analysed by Western blotting. (B) (C) Protein quantification of (A) by ImageJ. *n* = 3 in each group. *p < 0.05, **p < 0.01, ***p < 0.001 versus Ctrl. ^#^ *p* < 0.05, ^##^ *p* < 0.01, ^###^ *p* < 0.001 versus siRNA. ^*p* < 0.05, ^^*p* < 0.01, ^^^*p* < 0.001 versus ^+^siMETTL3. *p* < 0.05, ^++^ *p* < 0.01, ^+++^ *p* < 0.001 versus Ctrl+*S. aureus*. ^&^ *p* < 0.05, ^&&^ *p* < 0.01, ^&&&^ *p* < 0.001 versus siRNA+ *S. aureus* . ^%^ *p* < 0.05, ^%%^ *p* < 0.01, ^%%%^ *p* < 0.001 versus siMETTL3 + *S. aureus* . siRNA, small interfering RNA; si‐METTL3, siRNA targeting METTL3; *S. aureus* . *Staphylococcus aureus*.

It has been reported that the inhibition of osteoarthritis through the control of autophagy and inflammatory cell death of chondrocytes can be achieved by butyrate [28]. We speculate that the decrease in METTL3 expression by butyrate in osteomyelitis affects osteogenic activity by regulating the autophagy of osteoblasts. In order to validate this hypothesis, we examined the expression of autophagy‐related proteins, including LC3B and p62 [29], in METTL3‐siRNA‐transfected MC3T3‐E1 cells treated with *S. aureus* for 7 days. Compared with the simple infection group, the expression of LC3B increased and the expression of P62 decreased in the siMETTL3 group cells, and the cells treated with 10 μg/mL butyrate exhibited a similar effect (Figures 8B,C). These findings suggest that butyrate may enhance the osteogenic activity of osteomyelitis induced by *S. aureus* through the METTL3‐autophagy axis.

## 4 Discussion

Osteomyelitis is a severe bone infection associated with high levels of inflammation and progressive bone destruction and loss [30]. *S. aureus* is the most common pathogen of osteomyelitis. The treatment of osteomyelitis is often affected by chronic recurrent episodes and the emergence of antibiotic‐resistant strains, such as methicillin‐resistant *S. aureus* [31, 32]. Osteoblasts are the main functional cell types involved in bone formation, serving as the basis for bone matrix synthesis, secretion and mineralisation. In the context of osteomyelitis, the bone remodelling process involving osteoblasts and osteoclasts undergoes alterations, leading to abnormal bone formation and heightened bone degradation [33, 34]. In this study, we used MC3T3‐E1 cells to evaluate osteoblast function, and the results showed that during *S. aureus* infection in MC3T3‐E1 cells, the expression levels of osteogenic markers RUNX2 and OCN were down‐regulated, indicating that bone formation was inhibited.

Several studies have demonstrated that the inhibitory effect of butyrate on osteoclasts is mediated by its regulation of HDAC activity, thereby directly controlling ganglion bone resorption [22, 35]. Another study found that calvaria incubated with 500 nM sodium butyrate exhibited increased secretion of ALP compared to untreated controls after 24 h, which was attributed to the inhibition of HDAC activity [36]. More recently, it has been demonstrated that butyrate can modulate bone synthesis metabolism by promoting the production of Wnt 10b by CD8+ T cells, which is mediated by regulatory T cells [35]. Hence, we speculate that butyrate may exert a significant influence on bone formation in cases of osteomyelitis, which was further investigated through experimentation. Adding 10 or 20 μg/mL butyrate to MC3T3‐E1 cells exposed to *S. aureus* infection consistently restored the expression of OCN and RUNX2, and rescued bone mineralisation (evaluated by ALP staining levels), indicating that a certain concentration of butyrate has certain benefits for the osteogenic and can reverse mineralisation defects induced by *S. aureus* in MC3T3‐E1 cells.

During the prolonged infection of *S. aureus* in MC3T3‐E1 cells, an up‐regulation of m6A methyltransferase METTL3 was observed, which exhibited an inverse relationship with osteogenic markers. Down‐regulation of METTL3 by RNA interference rescued the decrease in osteogenic markers caused by infection. Simultaneously, the reduced expression of METTL3 was observed upon treatment of 10 to 20 μg/mL butyrate to MC3T3‐E1 cells exposed to *S. aureus* infection. The impact of butyrate treatment closely resembled that of silencing METTL3. Consequently, it can be inferred that prolonged *S. aureus* infection results in the up‐regulation of METTL3, subsequently leading to osteogenesis and bone mineralisation impairments. The utilisation of a specific concentration of butyrate or the suppression of METTL3 effectively rescued the osteogenic deficiencies.

To further validate our in vitro results, we employed a mouse model of *S. aureus* ‐induced osteomyelitis to simulate the process of human infection. We observed an elevation in local METTL3 levels in infected bones, prompting us to investigate whether the effectiveness of butyrate and siMETTL3 in mitigating osteogenic and mineralisation deficiencies in vitro could be replicated in vivo. Consequently, we administered pretreatment of butyrate and siMETTL3 to mice, followed by infection with *S. aureus* , and assessed the expression of various indicators in the bone marrow. The results demonstrated that pretreatment of *S. aureus* ‐infected mice effectively suppressed the up‐regulation of METTL3 and prevented the decrease in osteogenic index. Prior research has indicated that butyrate has the ability to modulate cellular autophagy and impact the progression of diseases [37, 38]. Therefore, we speculate that the beneficial effect of butyrate observed in osteomyelitis infection caused by *S. aureus* may be mediated through the METTL3‐autophagy axis. Our subsequent research also briefly validated this hypothesis. The expression of autophagy marker LC3B decreased in MC3T3‐E1 cells exposed to *S. aureus* infection, while the expression of p62, a protein regulating autophagy clearance of dysfunctional organelles or aggregates, increased, and butyrate or siMETTL3 treatment could reverse this change. This may explain why inhibiting the expression of METTL3 (whether it pertains to butyrate or siMETTL3) only enhances the osteogenic activity of infected cells, but not the uninfected cells. In cells not infected with *S. aureus* , autophagy was not impaired; therefore, inhibiting METTL3 expression to enhance autophagy may not lead to an increase in osteogenic activity. However, further research is needed to confirm the relationship between autophagy of osteoblasts and osteogenic activity. In addition, our experiment investigated the potential role of butyrate in osteomyelitis, with further evidence required to reveal the role of other short‐chain fatty acids in osteomyelitis treatment, especially acetate and propionate.

In conclusion, this study aims to investigate the roles of butyrate and METTL3 in the osteogenesis of osteomyelitis. Cell experiments show that *S. aureus* infection results in increased expression of METTL3 in osteoblasts, which subsequently leads to osteogenic and mineralisation impairments. Supplementing with a specific concentration of butyrate can reverse this phenomenon by inhibiting METTL3 expression. Furthermore, pretreatment with butyrate or siMETTL3 in a mouse osteomyelitis model can lead to an increased expression of osteogenic factors. These findings indicate that the m6A methyltransferase METTL3 plays a significant role in the pathogenesis of osteomyelitis, suggesting that butyrate holds potential as a future replacement therapy for this condition.

## Author Contributions

**Chongkai Sun:** conceptualization (equal), data curation (equal), investigation (equal), methodology (equal), writing – original draft (equal), writing – review and editing (equal). **Yuan Xu:** investigation (equal), visualization (equal). **Ziyue Peng:** data curation (equal), formal analysis (equal). **Xuyou Zhou:** investigation (equal), software (equal). **Zixuan Wang:** investigation (equal), validation (equal). **Haoyang Wan:** investigation (equal), methodology (equal), project administration (equal), resources (equal), supervision (equal), validation (equal). **Bin Yu:** conceptualization (equal), funding acquisition (equal), investigation (equal), methodology (equal), resources (equal), supervision (equal), writing – original draft (equal), writing – review and editing (equal).

## Conflicts of Interest

The authors declare no conflicts of interest.

## Data Availability

The data that support the findings of this study are available on request from the corresponding author. The data are not publicly available due to privacy or ethical restrictions.
